# Social Media Use and Loneliness Among Older Adults: The Mediating Role of Social Contact and Perceived Social Support

**DOI:** 10.1093/geroni/igaa057.1028

**Published:** 2020-12-16

**Authors:** Kunyu Zhang, Jeffrey Burr, Kyungmin Kim, Nina Silverstein, Qian Song

**Affiliations:** 1 University of Massachusetts Boston, Woburn, Massachusetts, United States; 2 University of Massachusetts Boston, Boston, Massachusetts, United States; 3 University of Massachusetts Boston, Needham, Massachusetts, United States

## Abstract

Loneliness is a risk factor for poor quality of life among older adults. Social media use provides a new dimension of communication for older adults to connect with people and to maintain social relationships. However, research has been inconclusive about whether social media use reduces loneliness among older adults, which is due in part to a lack of appropriate measures for capturing different types of social media use. Furthermore, little is known about the underlying mechanisms through which social media use is associated with loneliness. This study investigates the association between social media communication with close social ties and loneliness among community-dwelling older adults (65+), and further examines the mediating role of social contact and social support in the association. Data from the 2014 wave of the Health and Retirement Study (HRS) are analyzed to address our research questions (N = 4,184). Path analyses are employed to examine the relationships among social media communication with close social ties (i.e., children, family, and friends), frequency of contact with social ties (i.e., phone, in-person contact, write/email), perceived social support from social ties, and loneliness (R-UCLA loneliness scale). The results show that a higher level of social media communication is associated with lower levels of loneliness through social contact and perceived social support. Moreover, the relationship between social media communication and perceived social support is partially mediated by social contact. These findings suggest that social media communication may be considered an intervention that may reduce loneliness among older people.

